# Influence of Deadwood, Tree‐Related Microhabitats, and Forest Structural Features on Saproxylic Arthropod Diversity

**DOI:** 10.1002/ece3.73600

**Published:** 2026-04-30

**Authors:** Mohammad Jamil Shuvo, Michael Wohlwend, Katrin Heer, Yoan Paillet, Gernot Segelbacher

**Affiliations:** ^1^ Chair of Wildlife Ecology and Management, Institute of Forest Sciences, Faculty of Environment and Natural Resources University of Freiburg Freiburg im Breisgau Germany; ^2^ Mayr‐Stihl Professorship for Forest Genetics, Faculty of Environment and Natural Resources University of Freiburg Freiburg im Breisgau Germany; ^3^ INRAE, Lessem Univ. Grenoble Alpes Grenoble France

**Keywords:** arthropods, deadwood, eDNA metabarcoding, retention forestry, tree‐related microhabitats (TreMs)

## Abstract

Forest composition and structural diversity play a key role in shaping habitat availability and biodiversity, particularly influencing the occurrence of saproxylic species associated with tree‐related microhabitats (TreMs). TreMs and deadwood are essential features of forest ecosystems, offering critical habitats for saproxylic arthropods that contribute to decomposition, nutrient cycling, and overall forest resilience. Understanding the complex interactions between forest structures, TreMs, and arthropod communities is crucial for biodiversity conservation and sustainable forest management. This study examines how forest composition, deadwood characteristics, and TreMs influence saproxylic arthropods' diversity in the Black Forest, Germany. Using environmental DNA (eDNA) metabarcoding, we assessed arthropod communities across 135 forest plots with varying degrees of retention. Our findings reveal that specific TreMs, such as cavities, mosses, and insect galleries, were associated with arthropod richness patterns, particularly for Collembola, Hemiptera, and Arachnida. Canopy closure emerged as the most consistent predictor of arthropod richness, while tree‐related microhabitats, lying deadwood volume, snag volume, and forest management intensity showed additional taxon‐specific associations. These findings reveal that species richness is associated with multiple ecological factors, highlighting the complexity of forest ecosystems. They underscore the need for forest management strategies that preserve deadwood and TreMs, enhance structural heterogeneity, and consider broader indicators of forest naturalness to effectively support arthropod biodiversity.

## Graphical abstract

We used environmental DNA (eDNA) metabarcoding across 135 temperate forest plots in Germany to examine how deadwood, tree‐related microhabitats (TreMs), and forest structure shape saproxylic arthropod communities. Structural equation modeling revealed that TreM richness and deadwood volume are key predictors of arthropod diversity, highlighting the role of structural complexity in maintaining forest biodiversity. Our findings provide a scalable framework for linking molecular biodiversity data with ecological indicators in managed forests.

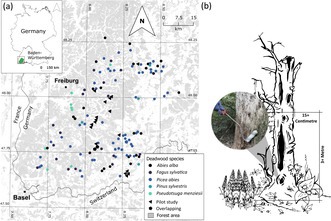

## 1 Introduction

Structural complexity in forest ecosystems underpins biodiversity by shaping habitat availability for numerous organisms (Seibold, Bässler, Baldrian, et al. 2016; Bauhus et al. 2018; Ehbrecht et al. 2017). Among these structural features, deadwood and tree‐related microhabitats (TreMs) have emerged as key elements influencing arthropod communities, particularly saproxylic species that rely on decaying wood or tree structures during their life cycles. Although these components differ in origin and form, together they contribute to habitat heterogeneity by offering complementary ecological functions that support species involved in decomposition, nutrient cycling, and forest resilience (Seibold, Bässler, Brandl, et al. 2016; Bauhus et al. 2018; Ulyshen and Šobotník 2018; Seibold and Thorn 2018; Majdi et al. 2025). Deadwood is typically defined as fallen logs, standing dead trees, stumps, or large branches in various stages of decomposition (Stokland et al. 2012; Bauhus et al. 2018; Lachat et al. 2013). These structures provide essential resources for saproxylic arthropods, which feed on, live in, or otherwise depend on decaying wood. As deadwood breaks down, it creates diverse habitats that attract a wide range of species, including wood‐boring beetles, fungal feeders, predators, and detritivores (Ulyshen 2016; Stokland et al. 2012; Wheater et al. 2023). In parallel, TreMs include features such as cavities, bark injuries, fungal growths, and epiphyte mats, which can form on both living and dead trees and often result from physical damage, decay, or biological activity (Bütler et al. 2020; Larrieu et al. 2018). While their ecological functions may overlap in some cases, TreMs and deadwood substrates represent distinct structural components of forest ecosystems. Together they increase habitat variety and harbor different, yet often complementary, arthropod communities (Stokland et al. 2012; Seibold, Bässler, Baldrian, et al. 2016; Bütler et al. 2020; Larrieu et al. 2018).

Deadwood contributes through trophic resources and structural variation, sustaining larger populations and promoting coexistence (Wright 1983; Srivastava and Lawton 1998; Clarke and Gaston 2006). Diversity increases with decay progression, from xylophagous beetles in early stages to detritivores, predators, and fungi‐associated specialists such as Ciidae in advanced stages (Seibold et al. 2015; Wheater et al. 2023; Stokland et al. 2012; Ulyshen 2016). Coarse woody debris, including logs, stumps, and snags, provides stable microclimates and long‐term habitat continuity, supporting more saproxylic species than smaller woody elements (Lachat et al. 2013; Bouget et al. 2013). TreMs complement this role by offering food, shelter, and nesting sites in otherwise uniform forests, and sustain unique specialist taxa across cavities, epiphytes, and bark features (Bütler et al. 2020; Larrieu et al. 2018; Bouget et al. 2013; Seibold, Bässler, Baldrian, et al. 2016).

Although the ecological significance of deadwood and TreMs is widely recognized, their combined influence on forest composition and management on arthropod diversity remains less understood. Previous studies examined general patterns of deadwood‐associated biodiversity (Seibold and Thorn 2018; Stokland et al. 2012; Seibold et al. 2015), but relatively few addressed how tree species, TreM types, deadwood heterogeneity, and retention practices jointly shape assemblages (Paillet et al. 2018). TreMs on both living and dead trees provide essential habitats, and dead trees in particular support higher TreM abundance and diversity, such as cavities and bark beetle galleries, benefiting numerous forest species (Asbeck et al. 2022; Paillet et al. 2019). Yet their availability has declined in managed forests due to intensive silviculture. German forests, long shaped by timber production, are now transitioning toward biodiversity‐oriented approaches (Storch et al. 2020). Retention forestry, which preserves habitat trees, snags, and logs, is considered effective for maintaining TreMs and deadwood (Gustafsson et al. 2012; Stokland et al. 2012; Storch et al. 2020). Mixed‐species stands further enhance habitat heterogeneity, resilience, and arthropod diversity (Oxbrough et al. 2012). Expanding retention areas and prioritizing diverse tree species and sizes have become central strategies for conservation (Großmann et al. 2023), though their effectiveness in sustaining saproxylic communities is still insufficiently resolved (Gustafsson et al. 2012; Storch et al. 2020; Großmann et al. 2023).

Understanding the ecological role of deadwood and TreMs in supporting terrestrial arthropods is essential for integrating biodiversity conservation into forest management. Arthropod communities have traditionally been studied with visual surveys, pitfall and interception traps, or sweep netting, approaches that are valuable but time‐consuming and taxonomically limited (Cohnstaedt et al. 2012). Advances in DNA‐based methods and high‐throughput sequencing (HTS) now allow more efficient biodiversity assessments of complex samples (Elbrecht et al. 2017; Buchner et al. 2021; Weber et al. 2024). Environmental DNA (eDNA) metabarcoding has become a powerful, non‐invasive tool to detect a wide range of arthropod taxa from substrates such as soil, litter, bark, or insect trap residues (Thomsen and Willerslev 2015; Ruppert et al. 2019; Krehenwinkel et al. 2022; Gamonal Gomez et al. 2023). The method is highly sensitive, avoids destructive sampling, and is particularly suited for sensitive forest systems (Macher et al. 2024; Yoneya et al. 2023; Kestel et al. 2024). A roller‐based surface eDNA approach recently proved effective for detecting TreM‐associated communities, especially detritivores and microarthropods (Shuvo et al. 2025), but its performance across taxa remains to be evaluated. Despite this potential, it has rarely been applied in large‐scale forest surveys. Here, we address this gap by applying the method across 135 Black Forest plots to assess its effectiveness and examine how forest structure, deadwood, TreMs, and management shape arthropod diversity in temperate forests.

We frame our study around three core hypotheses: (1) Saproxylic arthropod richness increases with forest structural complexity and is positively associated with canopy closure, deadwood availability (lying deadwood and snags), and higher levels of structural retention. (2) The presence and diversity of specific TreMs, particularly cavities, bark injuries, fungal growths, and epiphyte mats, are positively associated with saproxylic arthropod richness and can serve as indicators of structurally favorable habitat conditions. (3) Higher structural complexity reflected in deadwood heterogeneity and TreMs diversity is associated with increased taxonomic diversity of saproxylic arthropods.

Gaining insight into these relationships allows us to pinpoint critical habitat features that support near‐ground arthropods and to develop more focused conservation measures. These findings offer practical guidance for forest managers on incorporating habitat retention into standard forest planning, particularly in working landscapes where conservation opportunities depend on management decisions.

## 2 Materials and Methods

### 2.1 Study Region and Plots

**FIGURE 1:**
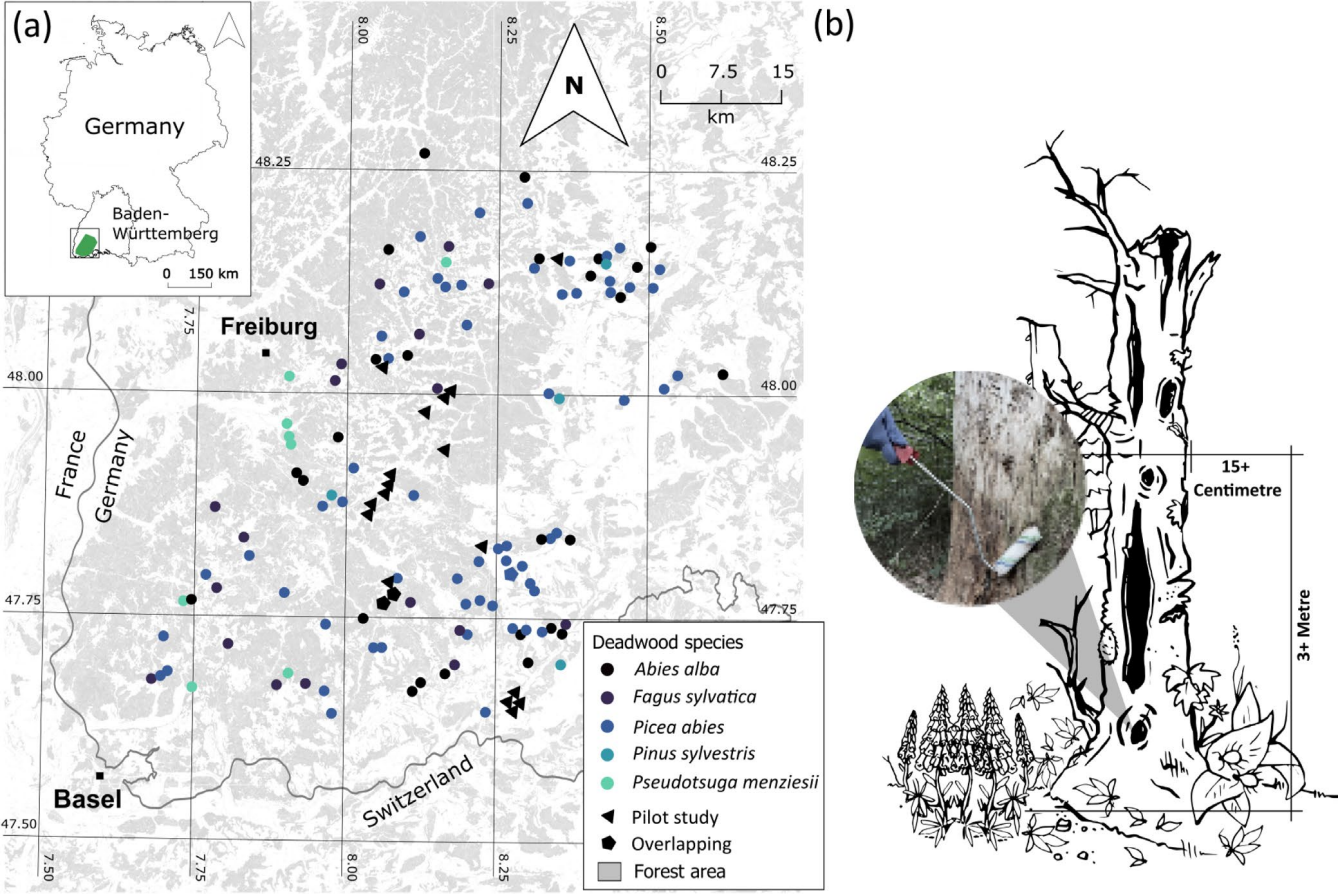
Map of 135 ConFoBi study sites in the Black Forest, Germany (Storch et al. 2020). (a) shows 20 pilot plots (triangles), 3 overlapping plots (hexagons), and 120 main plots (circles), with colors indicating sampled deadwood species (see legend). (b) illustrates the roller‐based eDNA sampling approach at a forest site.

This study was conducted in 135 one‐hectare plots of the ConFoBi project (established in 2016) in the southern Black Forest, Germany (Figure 1). The area spans > 5000 km^2^ of mixed forests dominated by Norway spruce ( *Picea abies* ), European beech ( *Fagus sylvatica* ), silver fir ( *Abies alba* ), and other common species including oak, maple, and Douglas fir. Plots range from 443 to 1334 m elevation with slopes up to 34° (Storch et al. 2020), and were selected to represent gradients in forest type, canopy cover, elevation, and deadwood, including unmanaged stands. Adjacent plots are spaced ≥ 750 m. For eDNA sampling, one standing dead tree of a locally dominant species (mainly spruce, fir, or beech) was chosen per plot (see Figure 1 for species distribution). The distribution of plots across forest and management types, as well as the distributions of key continuous environmental variables (elevation, canopy closure, and deadwood characteristics), are summarized in the (Table S1 and Figure S2). The ConFoBi plots span gradients of forest management intensity and structural complexity, including variation in tree species composition, canopy closure, deadwood amount and type, and tree‐related microhabitat availability (Storch et al. 2020).

### 2.2 Sample Collection and Processing

Field samples were collected in two batches. The first 20 samples were collected in April–May 2023 as a pilot study for methodology development (Shuvo et al. 2025). The main sampling in August–September 2023 covered the remaining 120 ConFoBi plots, including 3 overlapping with the pilot study. Analyses were conducted on both sampling batches to assess methodological consistency; however, all statistical analyses and result interpretations presented in this study are based exclusively on the 120 main samples to avoid seasonal bias. In each plot, one standing dead tree representing the dominant species within 50 m of the center point was sampled, with DBH ≥ 15 cm, height ≥ 3 m, and classified by decay stage. If several trees qualified, the closest to the center point was selected. Species representation varied across the landscape: *Picea abies* (*n* = 60), *Abies alba* (*n* = 26), *Fagus sylvatica* (*n* = 18), *Pseudotsuga menziesii* (*n* = 8), and *Pinus sylvestris* (*n* = 4). This captured ecological variability but led to unequal sample sizes, which were accounted for statistically. TreMs up to breast height were identified using established catalogs (Bütler et al. 2020). Sampling was conducted along the trunk from the base of the tree up to breast height, with repeated passes covering a continuous vertical bark section and all accessible TreMs within this zone, resulting in an integrated surface sample rather than point‐based collection. Environmental DNA (eDNA) was collected by rolling a sterile 10 cm paint roller moistened with distilled water across bark, cavities, and other TreM surfaces (Shuvo et al. 2025). The roller‐based surface eDNA approach targets arthropod taxa that interact with bark surfaces and tree‐related microhabitats, including saproxylic, detritivorous, and surface‐active groups. As demonstrated in the methodological comparison by Shuvo et al. (2025), this approach effectively captures TreM‐associated communities while under‐representing highly mobile canopy taxa or organisms primarily inhabiting soil and litter layers. Separate rollers were used per tree. The roller was shaken in a sterile bag with distilled water, and debris was transferred by rubbing for ~30 s. During roller application, cavity surfaces were sampled as deeply as physically accessible, with the roller introduced into cavities wherever cavity size and shape allowed; however, access to deeper interior surfaces was limited for small or narrow cavities due to roller dimensions. Cavity presence and associated tree‐related microhabitat metadata were recorded following standardized TreM inventories, while detailed cavity morphometrics were not the primary focus of the surface‐based eDNA sampling. Samples were filtered with a 50 mL sterile syringe and a 0.45 μm polycarbonate track‐etched (PCTE) membrane, then preserved in buffer, sealed with luer caps, and stored at room temperature for up to 7 days before DNA extraction (IdentMe GmbH, Halle, Germany). To prevent contamination, sterile gloves were worn and changed between samples.

### 2.3 DNA Extraction, PCR Amplification, and Sequencing

DNA was extracted by IdentMe using a modified DNeasy Blood and Tissue Kit (Qiagen, Hilden, Germany) following established protocols (Miya et al. 2016; Hausmann et al. 2020). Extracted DNA was transferred to AIM (Leipzig, Germany) for metabarcoding analysis. For each sample, 5 μL of genomic DNA was amplified with Plant MyTaq (Bioline, Luckenwalde, Germany) using mini‐barcode primers targeting the mitochondrial *COI* region (313 bp; dgLCO1490/dgHCO2198, Leray et al. 2013). PCR amplification followed standard cycling conditions, including an initial denaturation step, 35 amplification cycles, and a final extension, using annealing temperatures consistent with established metabarcoding protocols (Leray et al. 2013; Shuvo et al. 2025). Amplicons were verified by gel electrophoresis, purified, and resuspended in molecular‐grade water. In a second PCR, Illumina Nextera XT indices (Illumina Inc., San Diego, USA) were ligated, and success was confirmed by gel electrophoresis. DNA concentrations were quantified with a Qubit fluorometer (Life Technologies, Carlsbad, CA, USA), and samples were pooled in equimolar amounts (100 ng each). Pools were purified with MagSi‐NGSprep Plus beads (Steinbrenner Laborsysteme GmbH, Wiesenbach, Germany) and sequenced on an Illumina MiSeq v3 platform (2 × 300 bp, 600 cycles, up to 25 million paired‐end reads).

### 2.4 Bioinformatic Processing

Sequences were processed with AIM's pipeline (Hausmann et al. 2020). Quality was assessed with FastQC v0.11.9, reads merged using Usearch v11.0.667 (Edgar 2010), primers trimmed with Cutadapt 3.5 (Martin 2011), and sequences (300–315 bp, ≤ 1 expected error) filtered with Vsearch v2.21.1 (Rognes et al. 2016). Dereplicated non‐singleton sequences were clustered into OTUs at 97% similarity, and chimeras were removed. A custom Perl script (AIM) retained non‐chimeric sequences and recovered quality‐filtered reads, including singletons. OTU tables were generated with SWARM v3.1.0 (Mahé et al. 2022). Taxonomic assignment used MegaBLAST against custom databases from GenBank (NCBI nucleotide) and BOLD, curated in Geneious v10.2.5 (Morinière et al. 2016), and the RDP classifier trained on arthropod/chordate *COI* datasets (Porter and Hajibabaei 2018). OTUs present in negative controls were removed if below the maximum control read counts. Consensus taxonomy was derived from BOLD, NCBI, and RDP; fungal OTUs were excluded for separate analysis. OTUs not resolved to at least the family level or non‐arthropods were discarded, and duplicate species were merged. Community composition analyses focused on arthropod families and orders typical of deadwood (Table S2; Bütler et al. 2020; Stokland et al. 2012). All analyses were conducted on both filtered and unfiltered datasets to assess dataset effects.

### 2.5 Variable Descriptions

We selected a set of environmental variables to test their effect on arthropod OTU richness, namely (1) forest structure (DBH mean, basal area, percentage of coniferous trees, and canopy closure), (2) forest management intensity, and (3) deadwood characteristics (lying deadwood volume, snags and deadwood decay stage), (4) tree‐related microhabitats (TreMs), and (5) average elevation (Table 1). Forest structure variables included mean DBH, basal area, coniferous share, and canopy closure. Management intensity was quantified with the Forest Management Intensity Index (ForMI; Kahl and Bauhus 2014), integrating harvested tree volume, non‐native tree species, and proportion of saw‐cut deadwood. Higher ForMI values indicate greater management intensity. Forest inventory (2016–2017) and deadwood surveys (2020) were used (Storch et al. 2020; Asbeck and Frey 2021). No interventions occurred in plots between data collection and the 2023 sampling, so relative differences among plots were assumed stable. Deadwood volume (m^3^) was calculated from lying and standing pieces (DBH > 7 cm, height ≥ 1.3 m) using standard formulas along V‐transects. Decay stage and TreMs were recorded to capture both the number and diversity of TreMs per tree (Bütler Sauvain et al. 2024). Tree‐related microhabitats (TreMs) were recorded at the tree level following standardized TreM inventories (Kraus et al. 2016; Bütler et al. 2020). Recorded TreMs included major microhabitat categories such as cavities (e.g., woodpecker cavities, trunk cavities, and insect galleries), bark‐related features (e.g., bark loss and injuries), wood‐decay features (e.g., exposed sapwood), epiphytic structures (e.g., bryophytes and lichens), and fungal fruiting bodies. For analyses, TreMs were summarized as total TreM richness per tree and as the presence of individual TreM types, corresponding to the TreM categories shown in Figure 4. TreM richness was used as a descriptive measure of the number of different microhabitat types present on a tree and does not assign equal weighting to individual TreM types, which were additionally analyzed separately. Forest composition was described by tree species richness and percentage of conifers, based on basal area from the 2017 inventory (Storch et al. 2020). Canopy openness was measured with 18 hemispherical photographs per plot using a Solariscope SOL300 at herb‐layer height (Ing. ‐Büro Behling, Wedemark). Structural complexity was quantified with the Stand Structural Complexity Index (SSCI) derived from terrestrial laser scanning, combining mean fractal dimension and effective number of layers across multiple plot positions (Ehbrecht et al. 2017; Frey et al. 2020). A summary of the minimum, maximum, and average values of all tested variables and units is provided in Table 1.

**TABLE 1:** Descriptive statistics (mean, SD, min, max) of variables on forest structure, deadwood, management intensity, and arthropod OTU richness across 120 plots.

Groups	Variable	Unit	Mean	Std	Min	Max
Forest structure	SSCI	Index	1.8	0.26	1.36	2.57
	DBHMean	mm	297.95	84.54	121.65	526.24
	Log (Basal area)	m^2^/ha	−5.72	0.3	−6.97	−4.92
	Percentage coniferous	%	0.72	0.25	0.08	1
	Canopy closure	Index	0.91	0.1	0.6	1
	Tree species	Richness	5.51	2.18	2	15
Average elevation		m	807.29	172.56	443	1334
Deadwood characteristics	Number of dead trees	Count/1 ha	34.41	57.03	0	394
	Sampled deadwood decaying stage	1–5 scale	1.78	0.98	1	4
	Snag volume	m^3^	7.28	14.99	0	142
	Lying deadwood volume	m^3^/ha	44.38	44.93	2.68	282.9
Microhabitats (TreMs)		Count per tree	5.02	1.46	2	9
Forest management intensity		Index	1.31	0.52	0.04	2.38
Arthropod taxonomic groups	Arachnida	OTUs richness	18.06	4.17	7	33
	Collembola	OTUs richness	4.8	1.72	1	9
	Diplopoda	OTUs richness	0.84	0.83	0	3
	Coleoptera	OTUs richness	5.69	2.85	1	15
	Hymenoptera	OTUs richness	1.64	1.48	0	9
	Hemiptera	OTUs richness	2.59	1.79	0	8
	Total richness	OTUs richness	78.02	34.37	22	214

### 2.6 Statistical Analysis

All statistical analyses were performed in R v4.1.1, with image adjustments in Inkscape v1.3.2. To check arthropod richness across deadwood species with unequal sample sizes, we applied rarefaction based on 1000 bootstrapped resamples, standardizing to the smallest group size (*n* = 4). Mean richness values were calculated per iteration, resulting in fractional estimates, using the vegan package (Oksanen et al. 2001). Predictor–response relationships were first explored using Pearson correlations, visualized with scatterplot matrices (GGally package, Schloerke et al. 2021). Variance Inflation Factors (VIF) were calculated to assess multicollinearity (car package, Fox et al. 2007). Generalized Additive Models (GAMs) were then fitted separately for each arthropod group using the mgcv package (Wood 2015; Wood 2017). Arthropod OTU richness was used as the response variable in each model, and predictor variables represented forest structure, deadwood availability, tree‐related microhabitats, management intensity, and elevation. GAMs were fitted using penalized spline smoothers, with smoothness parameters estimated by restricted maximum likelihood to allow flexible but controlled non‐linear responses.

## 3 Results

### 3.1 Taxonomic Composition of Arthropod Communities

**FIGURE 2:**
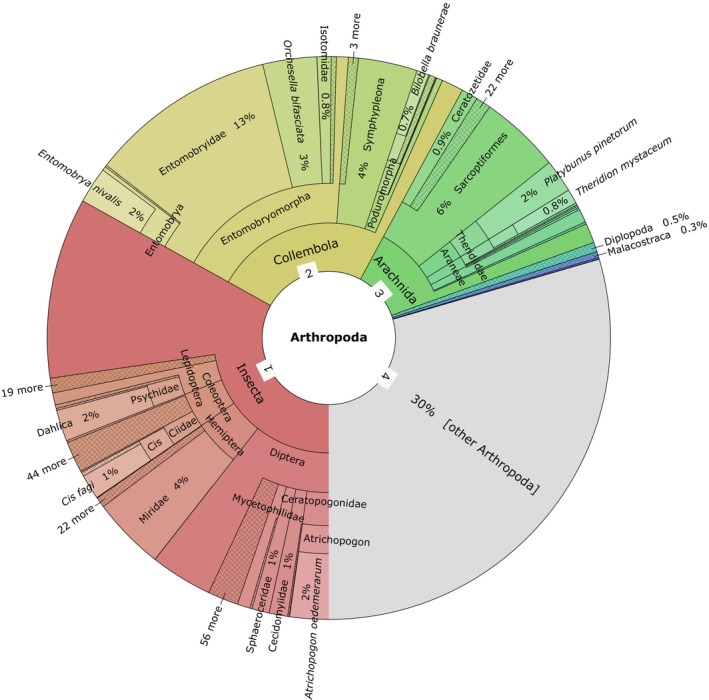
Taxonomic composition of arthropod OTUs in the TreMs dataset. The concentric circle shows classes, orders, and families from center to outer ring: Insecta (reddish shades) 45%, Collembola (yellowish) 25%, Arachnida (greenish) 15%, Diplopoda 0.5%, and remaining Arthropoda (gray) 30%.

Metabarcoding of 120 eDNA samples collected from deadwood structures containing tree‐related microhabitats (TreMs) produced 7,372,587 paired‐end reads (14,745,174 sequences) prior to quality filtering. After merging paired‐end reads, trimming primers, and applying quality filters, 6,256,624 sequences remained. Pre‐clustering and de novo chimera detection resulted in 11,066 operational taxonomic units (OTUs), of which 9661 were matched to the GenBank databases. Among the identified eukaryotes, 58% were classified as Ascomycota, 8% as Basidiomycota, and 18% as Arthropods. A total of 4073 unique OTUs were assigned to arthropods, with 910 OTUs retained after quality filtering for > 97% similarity and removal of duplicate species. Among these, the composition and relative abundance of arthropod taxa revealed that Insecta was the most dominant class, accounting for approximately 45% of the total taxa. Within this class, Diptera, Hymenoptera, and Coleoptera were the major contributing orders. Collembola represented a significant portion, contributing around 25% to the overall taxa distribution. Overall, Entomobryomorpha was the dominant Collembola order in the dataset, with *Entomobrya nivalis* and *Orchesella bifasciata* identified respectively at 2% and 3% of the total distribution. Arachnida was also present overall at about 15%, including the Araneae and Acari orders. Less dominant classes, such as Diplopoda and Malacostraca, collectively accounted for less than 1% of the taxa. Additionally, about 30% of the total taxa remained unassigned to specific arthropod groups or had a minimal share in the total OTU count and were categorized as “Other Arthropoda” (Figure 2). After an additional round of filtering to identify TreMs‐associated arthropods (Bütler Sauvain et al. 2024; Bütler et al. 2020; Stokland et al. 2012), we identified 55 families across different taxonomic classes and orders. Because OTU clustering at a fixed similarity threshold may introduce bias for taxonomically poorly resolved groups such as Collembola and Acari, richness and composition patterns are interpreted at the family and order level. Coleoptera and Diptera dominated among insects, while Entomobryomorpha contributed the most within Collembola, and Mesostigmata was the most abundant order within Arachnida. In total, we identified 18 arthropod orders across five taxonomic classes. Additionally, the species accumulation curves demonstrated the effectiveness of the sampling effort in capturing taxonomic richness (Figure S1). In all groups, richness increased as more samples were added, though the number of taxa varied by group and taxonomic level. Coleoptera and Arachnida showed the highest numbers of detected species, with Coleoptera also having the most genera and families. Hymenoptera and Hemiptera showed similar trends but with fewer taxa overall. Collembola showed moderate richness across all levels, while Diplopoda had the lowest number of detected taxa and did not reach a plateau in the curves.

### 3.2 Arthropod Richness and TreMs Association Across Deadwood Species

**FIGURE 3:**
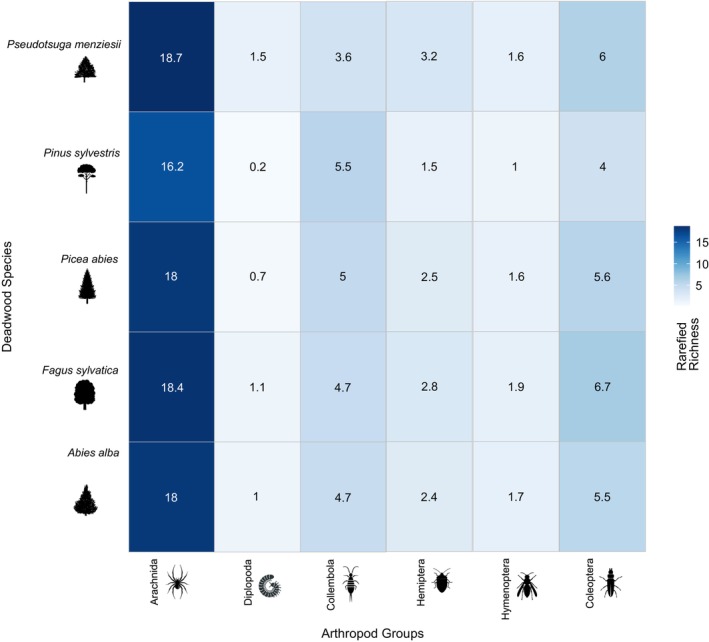
Rarefied arthropod family richness across deadwood species. Values were standardized by resampling to equal sample size (*n* = 4, 1000 iterations) and represent expected richness at this sampling effort, not asymptotic richness estimates. Darker blue indicates higher richness.

**FIGURE 4:**
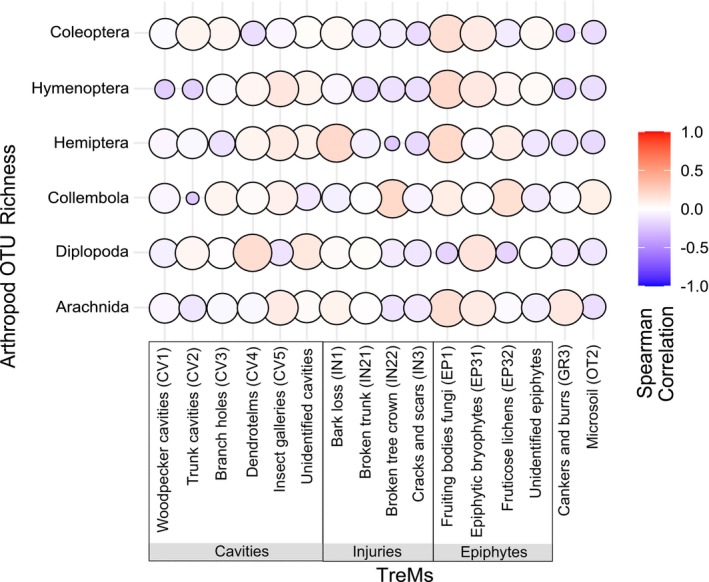
Spearman correlation between TreM types and arthropod OTU richness. Circle size reflects correlation strength; color shows direction (red = positive, blue = negative). TreM types on the x‐axis follow Kraus et al. (2016), and arthropod groups are on the y‐axis.

Arthropod richness varied across deadwood species, based on rarefied estimates that reflected expected family‐level diversity under equal sampling effort (see Methods). *Pseudotsuga menziesii* and *Fagus sylvatica* supported the highest overall richness at the family level, with Arachnida being the most diverse group (18.7 and 18.4 families, respectively), followed closely by *Abies alba* and *Picea abies* (both at 18.0). In contrast, *Pinus sylvestris* exhibited the lowest richness across most groups, including Arachnida (16.2 families). Among arthropod groups, Arachnida consistently showed the highest richness across all tree species. Coleoptera richness was highest in *Fagus sylvatica* (6.7 families), followed by *Pseudotsuga menziesii* (6.0) and *Picea abies* (5.6), while *Abies alba* also supported moderate richness (5.5). Collembola richness remained relatively stable across species, ranging from 4.7 to 5.5 families. Diplopoda richness was generally low, with the highest value observed in *Pseudotsuga menziesii* (1.5 families) and minimal representation in *Pinus sylvestris* (0.2 families) (Figure 3). Consistent with these descriptive patterns, a permutation‐based test using plot‐level arthropod richness did not detect a significant association between deadwood tree species and total arthropod family richness (*F* = 1.22, *R* ^2^ = 0.04, *p* = 0.32). Tree species identity was not independent of other forest structural variables, such as deadwood characteristics and stand structure, which were examined explicitly in subsequent multivariate analyses. Various taxonomic groups of arthropods also displayed variation based on the availability of different TreM types across deadwood species. TreMs were recorded at the tree level, with each sampled dead tree visually assessed for microhabitat features following the field catalog by Kraus et al. (2016). Among all observed structures, insect galleries, bark loss, and epiphytic bryophytes were among the most frequently encountered across the plots. Several TreM exhibited significant correlations with arthropod richness, varying across different arthropod groups. Collembola and Hemiptera were more frequently found in trees with woodpecker cavities, epiphytic bryophytes, and insect galleries. More Collembola were also found on exposed sapwood, while fewer were present in trunk cavities. Hemiptera were commonly associated with fruticose lichens and other epiphytic structures. Diplopoda displayed generally weak patterns across all TreMs, suggesting broader tolerance to habitat features. Arachnida and Hymenoptera were more often found in trees with woodpecker cavities and barkless areas but occurred less often in trees exhibiting bark loss, trunk cavities, or broken trunk injuries (Figure 4).

### 3.3 Influence of Forest Structure, Management, and Deadwood Characteristics on Arthropod Groups

**FIGURE 5:**
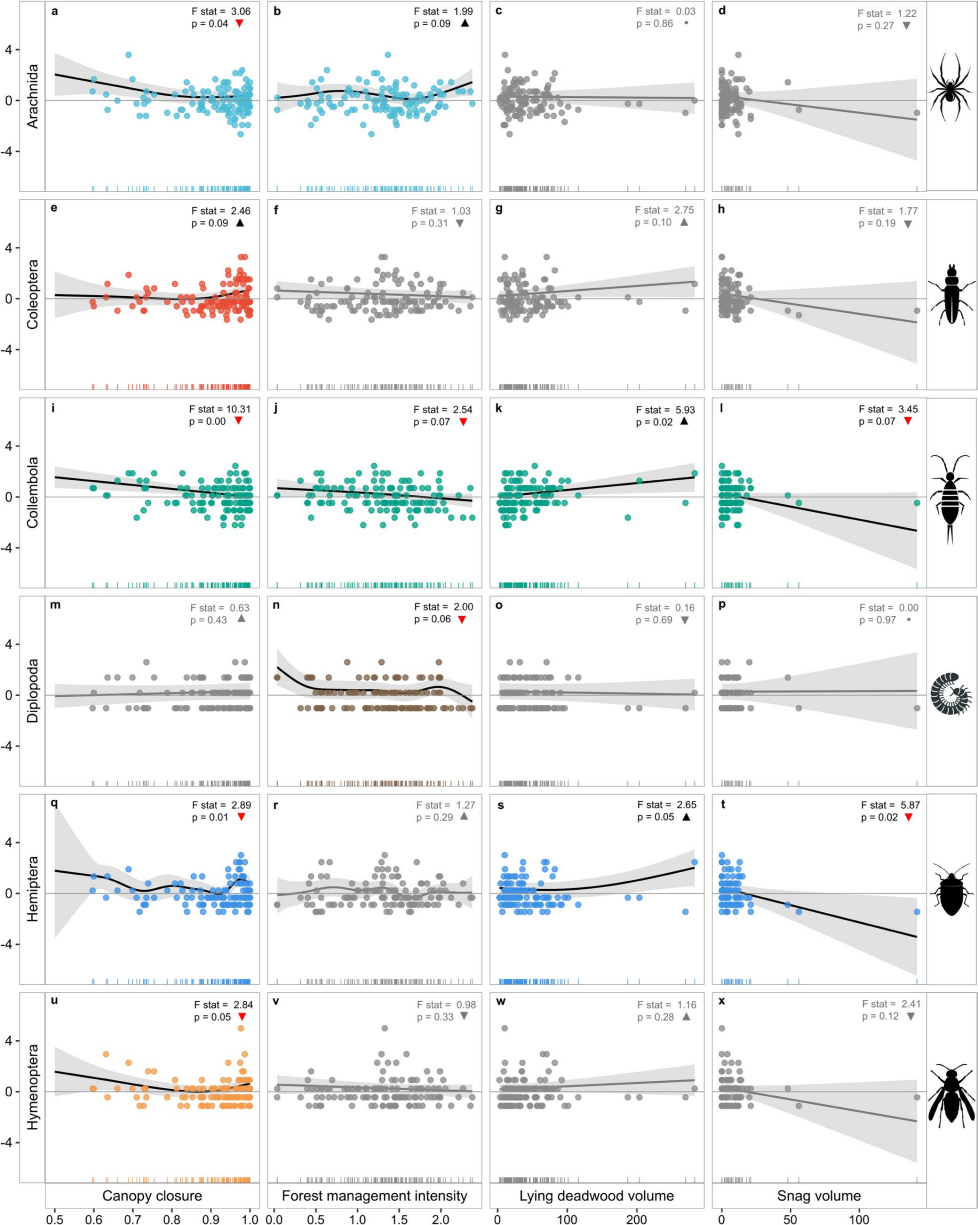
Generalized Additive Model (GAM) partial relationships between selected forest habitat variables and arthropod OTU richness across taxonomic groups. Panels show the fitted partial smooths for four focal predictors selected for visual summary based on the study's main ecological hypotheses: Canopy closure, forest management intensity, lying deadwood volume, and snag volume. Rows correspond to arthropod groups, and points represent observed standardized richness values. Solid lines indicate fitted GAM smooths and shaded bands represent 95% confidence intervals. Colored panels denote statistically supported relationships, whereas gray panels indicate non‐significant smooth terms. *F*‐statistics and *p*‐values are shown within panels. Upward (black) and downward (red) symbols indicate the overall direction of the fitted relationship across the observed gradient. Full GAM results for all tested predictors are provided in Table 2.

Forest structure, management intensity, and deadwood characteristics varied across plots (Table 1), with limited multicollinearity (Figure S2). Strong correlations occurred between lying deadwood volume and snags (*r* = 0.82) and between TreMs and dead tree counts (*r* = 0.85). DBH mean correlated positively with elevation and negatively with canopy closure. Among arthropod groups (Figure S3), Coleoptera correlated with Hymenoptera and Hemiptera, while species richness increased with Hymenoptera and Hemiptera. Arachnida showed a weak negative correlation with Diplopoda. Generalized Additive Models (Table 2) revealed both shared and taxon‐specific associations between arthropod OTU richness and habitat variables. Canopy closure was the most consistently supported predictor, showing associations with Hymenoptera, Hemiptera, Collembola, and Arachnida, and a marginal association with Coleoptera. Tree‐related microhabitats (TreMs) were positively associated with Hymenoptera, Diplopoda, and Arachnida. Lying deadwood volume and snag volume were particularly relevant for Hemiptera and Collembola, although support differed between predictors, with snag volume significant for Hemiptera and lying deadwood volume significant for Collembola, while the corresponding other associations were marginal. Diplopoda showed additional associations with DBH mean, basal area, percentage of coniferous trees, and SSCI, indicating sensitivity to broader stand structural conditions. Forest management intensity showed weaker but recurring associations with Arachnida, Collembola, and Diplopoda. For Coleoptera, only marginal associations were detected, mainly with canopy closure and percentage of coniferous trees (Table 2 and Figure 5).

**TABLE 2:** Results of Generalized Additive Models (GAMs) testing associations between arthropod OTU richness and forest structure, deadwood, microhabitat, and management variables.

Response variable	Predictor variable	edf	*F*‐statistic	*p*
Coleoptera	Canopy closure	2.22	2464	0.087
	Percentage of coniferous trees	1	2932	0.09
Hymenoptera	Tree‐related microhabitats (TreMs)	1	4341	0.04*
	Canopy closure	2.39	2839	0.049*
Hemiptera	Canopy closure	7.21	2.89	0.007**
	Snag volume	1	5871	0.018*
	Lying deadwood volume	1.79	2645	0.054
	Tree species richness	2.13	2691	0.055
	Basal area	1.2	3.53	0.095
	Percentage of coniferous trees	1	2811	0.097
Collembola	Canopy closure	1	10.312	0.002**
	Lying deadwood volume	1	5929	0.017*
	Stand Structural Complexity Index (SSCI)	1.71	3793	0.023*
	Snag volume	1	3453	0.066
	Forest management intensity	1.21	2544	0.071
	Tree‐related microhabitats (TreMs)	1.7	2389	0.073
Diplopoda	DBH mean	8.13	3169	0.002**
	Tree‐related microhabitats (TreMs)	1	7816	0.006**
	Basal area	1	7401	0.008**
	Percentage of coniferous trees	2.95	3.79	0.009**
	Stand Structural Complexity Index (SSCI)	3.42	2627	0.035*
	Forest management intensity	5.73	2	0.056
Arachnida	Tree‐related microhabitats (TreMs)	1	9118	0.003**
	Canopy closure	2.09	3063	0.039*
	Forest management intensity	3.97	1991	0.09

Significance levels are indicated as follows: * *p* < 0.05, **p < 0.01. Bold values correspond to statistically significant predictors (*p* < 0.05).

## 4 Discussion

This study aimed to understand how forest composition and structural features, particularly deadwood and tree‐related microhabitats (TreMs), shape arthropod biodiversity across managed temperate forests. Our findings showed that specific TreM types, such as cavities and epiphytic bryophytes, were associated with arthropod richness patterns across several groups, and that canopy closure was the most consistent predictor, while lying deadwood volume and snag volume showed additional taxon‐specific associations. We also observed substantial differences in arthropod diversity among tree species, with *Abies alba* and *Pseudotsuga menziesii* supporting the highest richness, while *Pinus sylvestris* consistently hosted the lowest. In terms of taxonomic groups, Insecta accounted for 45% and Collembola for 25% of all detected OTUs across plots, reflecting both their numerical dominance in near‐ground forest habitats and higher detectability of these comparatively well‐studied groups. The influence of forest structure, particularly canopy closure and deadwood characteristics, emerged as a central factor in shaping arthropod assemblages, while higher forest management intensity was associated with reduced OTU richness in several arthropod groups and with variation in structural habitat elements such as snags and TreMs.

### 4.1 Arthropod Composition and Habitat Preferences Across Forest Types

The analysis of arthropod communities revealed substantial diversity in taxonomic composition, with Insecta dominating the group and accounting for approximately 45% of all identified taxa. This finding aligned with previous research on arthropod communities associated with deadwood and TreMs, where insects such as Diptera, Hymenoptera, and Coleoptera comprised a major portion of arthropod communities (Shuvo et al. 2025; Bütler Sauvain et al. 2024). These groups play crucial ecological roles in pollination, decomposition, and predation, thereby enhancing forest biodiversity and ecosystem functioning (Stokland et al. 2012). Collembola represented around 25% of the total taxa, reinforcing its key role as a detritivore group that facilitated nutrient cycling and organic matter decomposition in both soil and decaying wood habitats (Bütler Sauvain et al. 2024). Arachnida, particularly the Araneae and Acari orders, also appeared frequently in the dataset, comprising 15% of the identified taxa. This pattern indicates a consistent association of arachnid taxa with standing deadwood and associated tree‐related microhabitats. Spiders, in particular, are frequently associated with deadwood, used tree trunks and logs as sites for web attachment and prey capture. However, while deadwood remained critical for many arachnid species, only a few qualify as strictly saproxylic (Buddle 2001). The low representation of Diplopoda and Malacostraca (less than 1%) likely reflected a combination of their generally lower abundance compared to insects, differences in detectability, and their tendency to occur primarily in soil and litter strata rather than on decaying wood (Stokland et al. 2012). Our observation suggests that the detected taxa in this study more commonly associate with wood surfaces, supporting the idea that different arthropod groups exhibited varying degrees of habitat specialization. In addition to these compositional trends, our findings revealed key limitations in species detectability and taxonomic resolution. Approximately 30% of the total taxa remained unassigned or had minimal representation, reflecting gaps in reference databases and challenges in detecting small or cryptic groups using eDNA. Species accumulation curves showed that Coleoptera and Arachnida were well captured in our sampling approach, while Collembola and especially Diplopoda continued to rise, suggesting under‐sampling. This likely reflects both their habitat preferences, particularly for soil and litter environments, and the sampling bias of the roller‐based method, which favors taxa on deadwood surfaces. While effective for surface‐active groups, this approach remains less suitable for those more loosely associated with wood substrates. These patterns highlight not only the completeness of sampling but also the ecological detectability of different arthropod groups. To improve future assessments, complementary methods targeting additional microhabitats should be considered.

Beyond taxonomic differences, arthropod richness also varied notably among deadwood tree species. *Pseudotsuga menziesii* and *Fagus sylvatica* supported the highest overall arthropod richness at the taxonomic family level, with Arachnida as the most diverse group across all species. Notably, *Abies alba* showed richness levels comparable to the top two species, underscoring its role as an ecologically favorable substrate. This contrasts slightly with our initial expectation that *Abies alba* would host the highest richness but aligns with emerging evidence that broadleaved species and non‐native conifers can provide complex and suitable microhabitats for a variety of arthropods. This finding aligned with previous studies indicating that coniferous tree species offer favorable conditions for a wide range of arthropod taxa, likely due to their structural properties, including bark texture and wood composition. In this context, deadwood of silver fir ( *Abies alba* ) supports comparatively high arthropod richness probably due to its persistent, fissured bark and relatively slow, moisture‐retaining decay processes, which promote fungal development and provide stable microhabitats for saproxylic and predatory taxa (Stokland et al. 2012; Spînu et al. 2024; Emrich et al. 2025). High richness of Coleoptera families observed in *Fagus sylvatica* may be linked to its smoother bark and faster decay dynamics, which enhance habitat suitability for wood‐associated beetles (Trappmann et al. 2013; Kahl et al. 2017). *Picea abies* also supported relatively high arthropod diversity, with particularly high richness observed in Arachnida and Coleoptera. This remained consistent with findings from other temperate forest studies, which showed that different tree species provided varying degrees of habitat suitability for different arthropod groups through different structural and chemical pathways (Bütler Sauvain et al. 2024). In contrast, *Pinus sylvestris* consistently exhibited the lowest overall arthropod richness across all taxa, including Arachnida, Coleoptera, and Diplopoda. This could have resulted from the higher resin content and different chemical composition of *Pinus sylvestris* , which may have deterred certain arthropod species from colonizing this substrate (Mumm and Hilker 2006). The chemical properties of tree species influenced the types of microorganisms and invertebrates that thrived in their decaying wood, with some tree species providing more favorable conditions for decomposers and predators than others (Stokland et al. 2012). The low richness of Diplopoda observed across all species remains notable and may reflect their specific microhabitat preferences or limited dispersal capacity in coarse woody substrates. At the plot level, permutation‐based analyses indicated that deadwood tree species alone did not explain a large proportion of variation in arthropod richness, suggesting that the observed species‐level differences are best understood in combination with structural attributes and tree‐related microhabitats rather than as isolated effects. This pattern reinforced the need for targeted research into how tree species traits shape the availability and quality of habitat for different arthropod groups in forest ecosystems.

### 4.2 Differential Responses of Arthropod Taxonomic Groups to TreMs Specificity

The availability and characteristics of specific TreMs significantly influenced arthropod distribution and richness. Several types, including woodpecker cavities, epiphytic bryophytes, fruticose lichens, and insect galleries, showed strong positive correlations, consistent with previous studies highlighting their role as critical resources for arthropods (Bütler Sauvain et al. 2024; Stokland et al. 2012; Larrieu et al. 2018). By contrast, features such as trunk cavities, bark loss, and broken trunk injuries were linked to lower richness in some groups, illustrating the complexity of microhabitat selection and the influence of interacting ecological factors. Collembola, primarily detritivores, exhibited a strong positive correlation with exposed wood, which favors microbial colonization and fungal growth and in turn supports detritivorous activity (Stokland et al. 2012). They also frequently occurred in bryophytes, lichens, and insect galleries, which provide additional food and moisture (Skubała and Marzec 2013). This strong preference suggests that Collembola distributions are closely tied to decomposition processes and microbial communities in deadwood ecosystems. Conversely, their reduced presence in trunk and mold cavities may result from limited microbial substrates in more sheltered environments. Hemiptera likewise correlated positively with TreMs such as woodpecker cavities, insect galleries, and epiphytic structures, where they likely benefit from diverse food sources (fungal hyphae, plant material) and protection from stressors (Bütler Sauvain et al. 2024). Their frequent occurrence in bryophytes and lichens further indicates that these features foster microenvironments rich in microbial life, which enhance Hemipteran feeding and reproduction (Larrieu et al. 2018; Stokland et al. 2012).

Diplopoda exhibited weak associations with most TreMs, indicating low dependence on specific microhabitats. Their tolerance of varying conditions may explain their broader distribution, while the lack of strong patterns suggests that their abundance is more influenced by decay stage or microclimatic factors such as moisture (Seibold et al. 2015; Puverel et al. 2019). Arachnida and Hymenoptera showed varied responses. Hymenoptera were frequently associated with barkless injuries, likely providing nesting substrates or hunting grounds, whereas Arachnida generally showed weak or neutral responses across most TreMs. These patterns are more parsimoniously explained by shared associations with exposed wood structures and specific TreMs, rather than by direct inference about functional roles within Hymenoptera. Both groups were less often linked to bark loss and trunk cavities, which are characterized by higher microclimatic exposure and reduced shelter. Similarly, weak associations with woodpecker cavities and broken trunk injuries indicate that cavity‐related structures differ in their relevance for different arthropod groups. Wood density may also shape TreM stability, with denser wood buffering moisture and temperature fluctuations (Seibold et al. 2015). However, exposed wood, while favorable for some taxa, correlated negatively with others, likely due to rapid drying and reduced microclimatic stability. Excessive sun and wind exposure can therefore limit habitat suitability for many saproxylic arthropods, reducing biodiversity. This underscores the importance of management practices that minimize deadwood exposure to extreme conditions (Ranius et al. 2024). Taken together, these results emphasize that TreM diversity and microclimatic conditions jointly shape arthropod communities, and that conserving a range of features under stable conditions is critical for biodiversity.

Fine‐scale structural features, such as insect galleries and bryophyte mats, enhance arthropod diversity by offering distinct resources and microclimates. This pattern supports ecological theories of niche differentiation and habitat filtering (Wright 1983; Seibold et al. 2015). Weak or inconsistent responses in Diplopoda likely reflect their ecology as moisture‐dependent detritivores with limited mobility, which makes them less tightly associated with specific tree‐related microhabitats than more mobile or structurally specialized arthropod groups. In contrast, clearer associations observed for Arachnida, Hymenoptera, and Hemiptera highlight the role of particular TreMs in providing shelter, hunting substrates, or resource access, indicating that taxon‐specific ecological traits shape arthropod responses to microhabitat diversity (Stokland et al. 2012; Seibold et al. 2015). Features such as woodpecker cavities, epiphyte mats, and barkless injuries consistently supported a broader range of taxa, while others, like trunk cavities and bark loss, were linked to reduced richness in certain groups. These findings suggest that maintaining a diversity of TreM types within forest stands can contribute to the conservation of saproxylic arthropods. Management strategies that preserve structural heterogeneity at the tree level, particularly by retaining a representative spectrum of TreMs, may therefore enhance the ecological value of deadwood habitats in production forests.

### 4.3 The Multifaceted Impact of Forest Structure and Deadwood Attributes

Generalized Additive Model (GAM) results showed that arthropod richness was associated with multiple dimensions of forest structure and deadwood availability, but that the strength and form of these associations differed among taxonomic groups. This overall pattern is consistent with our first and third hypotheses, which predicted that saproxylic arthropod richness would respond to structural complexity, deadwood availability, and microhabitat heterogeneity. Among the tested predictors, canopy closure emerged as the most consistent correlate of arthropod richness across groups. It was significantly associated with Hymenoptera, Hemiptera, Collembola, and Arachnida, and showed a marginal association with Coleoptera. This broad pattern suggests that canopy structure is a central component of habitat suitability in these forest systems, likely because canopy closure influences humidity stability, temperature buffering, and desiccation risk near deadwood surfaces, which are known to affect arthropod communities directly and indirectly through fungal, microbial, and detrital resources (Seibold, Bässler, Brandl, et al. 2016; Wu et al. 2021). Deadwood‐related predictors also played an important role, particularly for Collembola and Hemiptera. Both lying deadwood volume and snag volume were associated with richness patterns in these groups, but the strength of support differed: lying deadwood was significant for Collembola and marginal for Hemiptera, whereas snag volume was significant for Hemiptera and marginal for Collembola. These results are ecologically plausible because standing and lying deadwood provide complementary habitat functions. Lying deadwood tends to retain moisture, support fungal development, and promote decomposition processes, whereas snags provide vertical structural complexity, bark surface heterogeneity, and shelter opportunities for more mobile and surface‐active taxa (Stokland et al. 2012; Ulyshen 2016; Seibold and Thorn 2018). The fact that both variables were relevant, but not equally across taxa, reinforces the view that deadwood quantity alone is insufficient and that the form and diversity of deadwood substrates are also important for sustaining saproxylic communities.

Tree‐related microhabitats (TreMs) remained another central component of the arthropod richness patterns observed here. In the GAMs, TreMs were significantly associated with Hymenoptera, Diplopoda, and Arachnida, and marginally associated with Collembola. This result fits well with our second hypothesis, which proposed that the presence and diversity of TreMs would be positively associated with saproxylic arthropod richness and would indicate structurally favorable habitat conditions. TreMs capture fine‐scale habitat variation that broader stand metrics cannot fully describe. Cavities, bark injuries, fungal structures, epiphytes, and exposed wood surfaces can each create distinct combinations of shelter, humidity, feeding substrate, and colonization opportunities, thereby contributing to local habitat heterogeneity (Bütler et al. 2020; Larrieu et al. 2018; Asbeck et al. 2021). Our results therefore support the interpretation that TreMs are not only descriptive biodiversity indicators, but also structurally meaningful habitat attributes associated with differences in arthropod richness. Diplopoda showed the broadest suite of significant associations among all response groups, including DBH mean, basal area, percentage of coniferous trees, SSCI, and TreMs, with an additional marginal association with forest management intensity. This suggests that Diplopoda may be especially responsive to combined gradients of stand structure and microhabitat availability. Their strong relationship with DBH mean and basal area indicates that larger‐tree and denser‐stand conditions may influence habitat suitability, possibly through moisture buffering, litter accumulation, and substrate continuity, while their response to conifer share and SSCI suggests sensitivity to broader forest structural context rather than only the local properties of the sampled deadwood tree. Because Diplopoda are relatively low‐mobility detritivores with strong moisture dependence, such broader structural associations are ecologically plausible and align with earlier indications that this group responds to microclimatic and substrate conditions rather than to individual TreM features alone (Seibold et al. 2015; Puverel et al. 2019).

By contrast, Coleoptera showed only marginal associations mainly with canopy closure and percentage of coniferous trees. This is notable because beetles are among the most frequently studied saproxylic taxa and are often strongly associated with deadwood characteristics (Stokland et al. 2012; Seibold and Thorn 2018). In the present dataset, the weaker Coleoptera signal may reflect the broad OTU‐richness approach, which combines taxa with different ecological strategies, as well as limitations of surface‐based eDNA sampling for taxa associated with deeper wood tissue or more internal microhabitats. It is therefore likely that beetle richness in this system depends on finer‐scale substrate properties, such as bark condition, fungal colonization, wood quality, and decay continuity, which were not fully resolved in the present models. Forest management intensity showed weaker but recurring associations with Arachnida, Collembola, and Diplopoda. Although these were not the strongest effects in the models, their repeated appearance across groups is consistent with the expectation that intensive management is associated with simplified habitat structure and reduced diversity of deadwood‐associated resources (Jonsson et al. 2016; Löfroth et al. 2023). In the present study, forest management intensity is best interpreted as an integrative descriptor of long‐term stand context rather than as a direct mechanistic driver. Its associations likely reflect linked changes in deadwood retention, snag continuity, stand openness, and microhabitat availability, which is in line with previous work showing that managed forests often support fewer structural elements important for deadwood‐dependent biodiversity, even where some retention features remain present (Großmann et al. 2023; Storch et al. 2020). Taken together, these findings reinforce the idea that saproxylic arthropod richness is shaped by overlapping but non‐identical habitat dimensions. Canopy structure, deadwood quantity and form, TreM availability, and broader stand structural conditions all contributed to richness patterns, but their relative importance differed among taxonomic groups. This supports a central message of the study: arthropod biodiversity in managed temperate forests reflects the combined influence of forest structure and tree‐level habitat complexity, with different taxa responding to different components of this structural mosaic. Because these analyses are based on observational data, the identified relationships should be interpreted as patterns of association rather than direct causal effects. Nonetheless, the consistency of several predictors across groups, especially canopy closure, deadwood availability, and TreMs, supports their ecological relevance and practical value for biodiversity‐oriented forest management.

### 4.4 Implications for Conservation and Management

Our results are consistent with previous studies reporting lower arthropod diversity in forest stands characterized by higher management intensity. Practices such as reducing snags and deadwood, shorter rotations, high harvest rates, and planting fast‐growing species simplify habitats and reduce biodiversity (Löfroth et al. 2023; Jonsson et al. 2016). This simplification limits structural diversity and key resources like deadwood and TreMs, which are essential for arthropod communities (Stokland et al. 2012; Bütler et al. 2020). Management strategies that maintain canopy closure and preserve microhabitats such as hollows and epiphytes can mitigate these effects, helping reduce harvesting impacts and conserve biodiversity (Stokland et al. 2012; Paillet et al. 2018). Our results also revealed research gaps. A key limitation was the focus on taxonomic richness, while functional diversity and ecological interactions were not fully explored. Functional traits such as feeding, reproduction, and interspecific interactions are vital for assessing ecosystem services (Elizalde et al. 2020). Although eDNA enabled broad taxonomic detection, functional roles such as saproxylic affinity were not assigned due to limited trait databases and incomplete OTU resolution. Future work should build on trait‐linked resources to integrate taxonomic and functional perspectives (Le Guillarme et al. 2023). Arthropods provide services such as nutrient cycling, soil aeration, and pest regulation, and functional analyses would clarify their roles in ecosystem stability. Some groups, particularly Collembola and Diplopoda, remain overlooked in surveys despite important ecological roles. Their low detection rates highlight the need for integrative approaches combining eDNA and traditional methods (Shuvo et al. 2025; Leclerc et al. 2023; Ruppert et al. 2019). To sustain arthropod biodiversity, management should retain both standing and lying deadwood, as each provides distinct microhabitats. Deadwood across decay stages supports different communities, from fungal‐rich logs to decomposing bark (Stokland et al. 2012). Forest managers should preserve diverse deadwood types and additional features such as hollows, cavities, and epiphytes (Šebek 2016). In the face of climate change, structurally complex, mixed‐species stands and retention forestry remain key to biodiversity and resilience (Großmann et al. 2023). By promoting deadwood, TreMs, and diverse stands, managers and policymakers can sustain arthropod biodiversity and ecosystem services, strengthening forest stability and productivity.

These results are interpreted within the observational and landscape‐scale scope of this study. We identify consistent associations between forest structure, deadwood attributes, tree‐related microhabitats, and saproxylic arthropod richness, which are evaluated as patterns of covariation rather than as evidence of direct causal relationships. Tree‐related microhabitats were assessed as presence‐based structural features across a heterogeneous set of trees, which limits direct comparisons among standardized microhabitat units. Forest management intensity represents an integrative descriptor of long‐term management context and is therefore interpreted in relation to its association with structural habitat elements rather than as a directly tested mechanistic driver. Along with revealing broad, structurally mediated patterns in saproxylic arthropod communities, this study demonstrates that a roller‐based surface eDNA approach can reliably capture ecologically meaningful biodiversity signals at the landscape scale. This provides a foundation for future, more targeted ecological and conservation studies.

## 5 Conclusions

Arthropod diversity is shaped by a complex interplay of forest composition, deadwood availability, and microhabitat features such as TreMs, reflecting the ecological diversity and specialization within arthropod communities. Our findings show that canopy structure, deadwood availability, and tree‐related microhabitat richness are important predictors of arthropod richness and distribution across temperate forest landscapes. Higher forest management intensity was associated with lower richness in several arthropod groups, highlighting the need for biodiversity‐friendly, closer‐to‐nature forestry practices (Larsen et al. 2022). Retaining diverse deadwood structures and promoting habitat complexity could mitigate these effects. Future research should integrate functional trait analyses and molecular techniques to enhance taxonomic resolution and improve conservation strategies. Implementing evidence‐based management practices would help sustain arthropod biodiversity, ensuring long‐term ecosystem stability and resilience.

## Author Contributions

**Mohammad Jamil Shuvo:** conceptualization (lead), data curation (lead), formal analysis (lead), investigation (lead), methodology (lead), visualization (lead), writing – original draft (lead). **Michael Wohlwend:** data curation (supporting), formal analysis (supporting), project administration (equal), writing – review and editing (equal). **Katrin Heer:** conceptualization (supporting), formal analysis (supporting), supervision (equal), writing – review and editing (equal). **Yoan Paillet:** data curation (supporting), formal analysis (supporting), validation (supporting), visualization (supporting), writing – review and editing (equal). **Gernot Segelbacher:** conceptualization (equal), data curation (supporting), formal analysis (supporting), funding acquisition (equal), project administration (equal), supervision (equal), writing – review and editing (equal).

## Funding

This work was supported by GRK 2123/1 TPX.

## Conflicts of Interest

The authors declare no conflicts of interest.

## Supporting information


**Figure S1:** Species accumulation curves for different taxonomic groups (a–f), showing the relationship between species richness and the number of samples collected. Curves represent richness at family (purple), genus (blue), and species (yellow) levels. The asymptotic trends indicate the completeness of sampling for each group.
**Figure S2:** Scatterplot matrix illustrating the relationships among environmental variables, deadwood characteristics, and tree species composition.Diagonal panels show variable distributions; lower panels depict pairwise scatterplots, and upper panels display Pearson correlation coefficients with significance levels (*p* < 0.05, *p* < 0.01, *p* < 0.001). This visualization allows assessment of both linear associations and data distributions among key habitat features.
**Figure S3:** Scatterplot matrix showing pairwise relationships among arthropod taxonomic groups OTUs richness and overall species richness across all samples. Diagonal panels display variable distributions; lower panels show scatterplots between groups, and upper panels indicate Pearson correlation coefficients with significance levels (*p* < 0.05, *p* < 0.01, *p* < 0.001).

**Table S1:** Frequency distribution of study plots across forest types and management categories. Forest types were classified based on the proportion of coniferous trees per plot (coniferous ≥ 80%, deciduous ≤ 20%, mixed 20%–80%). Management categories were derived from the Forest Management Intensity index (ForMI; low < 0.33, medium 0.33–0.66, high > 0.66).
**Table S2:** Comprehensive overview of the identified tree‐related microhabitat (TreMs) associated arthropods at the taxonomic order, family, and species levels.

## Data Availability

The datasets generated and analyzed during the current study are available in the Figshare repository at https://doi.org/10.6084/m9.figshare.30421744. The repository includes the processed environmental DNA (eDNA) dataset, forest structural and arthropod diversity metadata, and all R scripts used for correlation analysis and statistical modeling.
