# Supplementary material for: Influence of Deadwood, Tree‐Related Microhabitats, and Forest Structural Features on Saproxylic Arthropod Diversity
Source: Ecol Evol. 2026 Apr 30;16(5):e73600. doi: 10.1002/ece3.73600 (PMC13129597; doi:10.1002/ece3.73600)
Supplement: Supplementary file 1 — Figure S1: Species accumulation curves for different taxonomic groups (a–f), showing the relationship between species richness and the number of samples collected. Curves represent richness at family (purple), genus (blue), and species (yellow) levels. The asymptotic trends indicate the completeness of sampling for each group. Figure S2: Scatterplot matrix illustrating the relationships among environmental variables, deadwood characteristics, and tree species composition.Diagonal panels show variable distributions; lower panels depict pairwise scatterplots, and upper panels display Pearson correlation coefficients with significance levels (p < 0.05, p < 0.01, p < 0.001). This visualization allows assessment of both linear associations and data distributions among key habitat features. Figure S3: Scatterplot matrix showing pairwise relationships among arthropod taxonomic groups OTUs richness and overall species richness across all samples. Diagonal panels display variable distributions; lower panels show scatterplots between groups, and upper panels indicate Pearson correlation coefficients with significance levels (p < 0.05, p < 0.01, p < 0.001). [file ECE3-16-e73600-s002.docx]

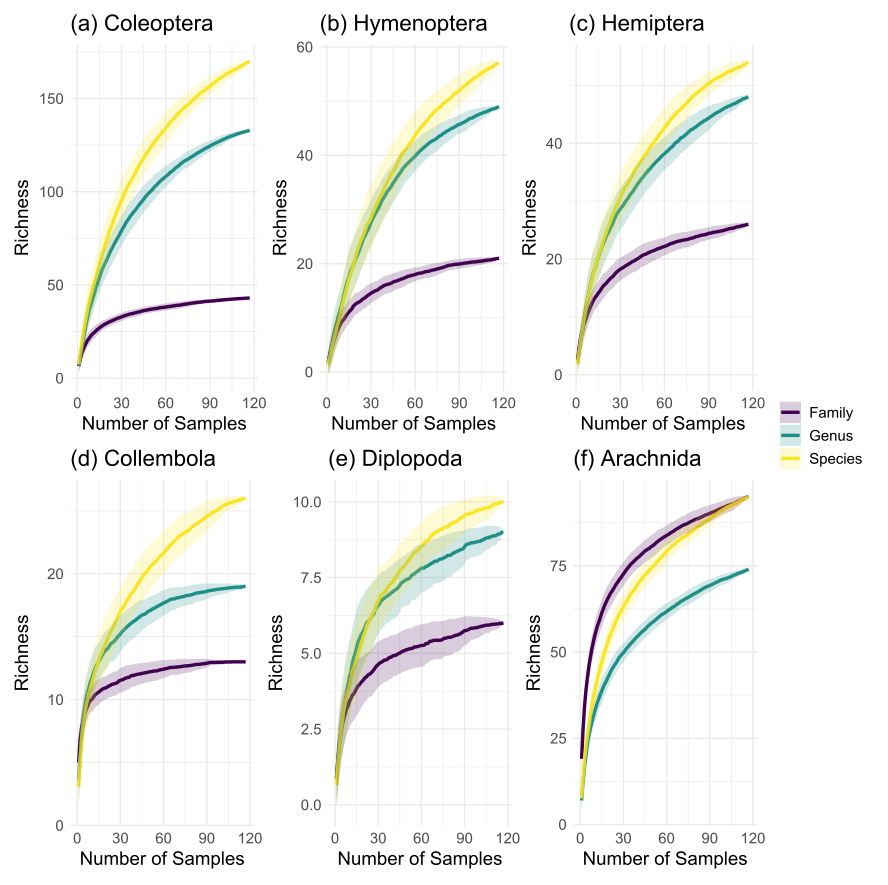


Figure S1. Species accumulation curves for different taxonomic groups (a–f), showing the relationship between species richness and the number of samples collected. Curves represent richness at family (purple), genus (blue), and species (yellow) levels. The asymptotic trends indicate the completeness of sampling for each group.


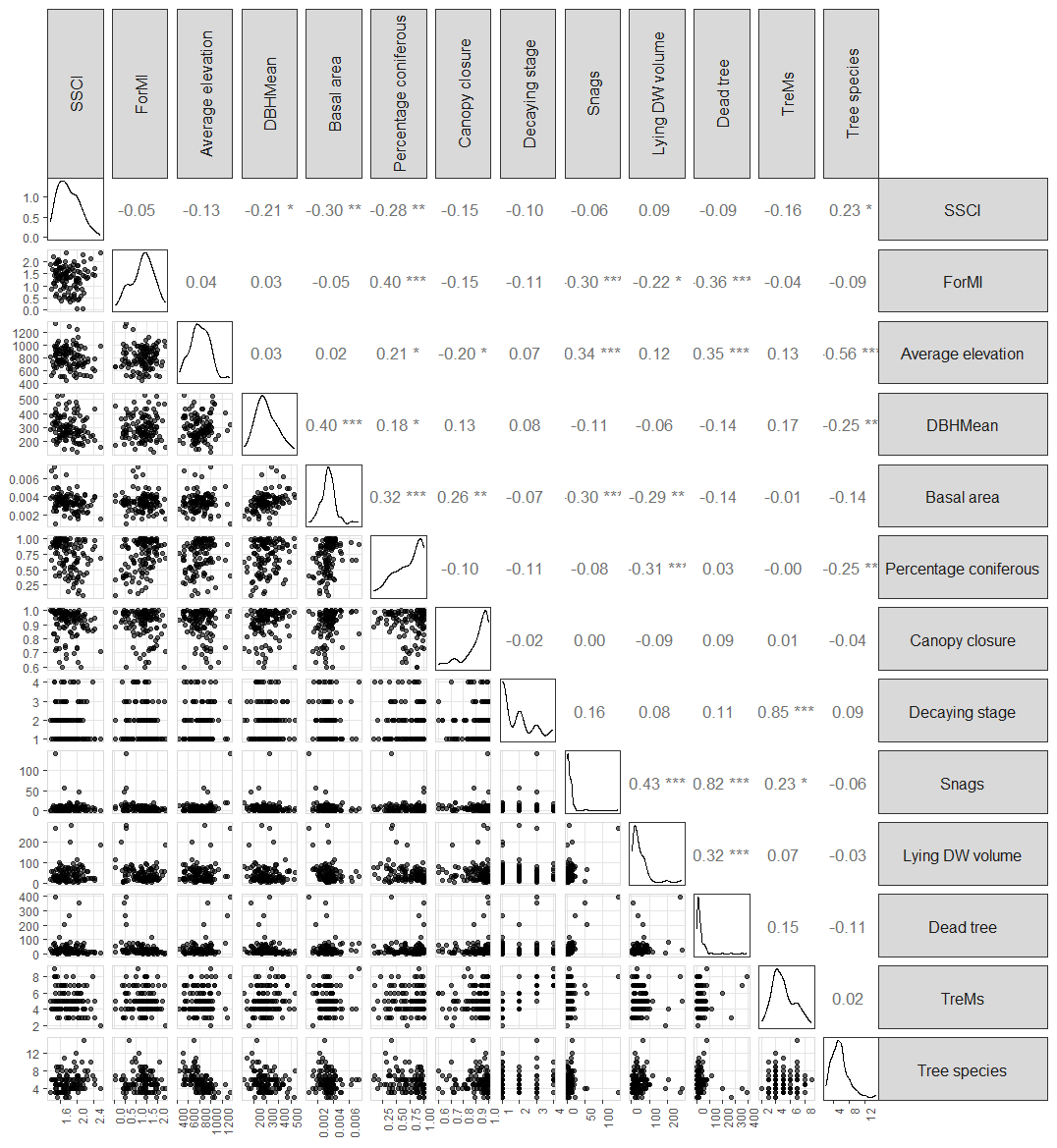


Figure S2. Scatterplot matrix illustrating the relationships among environmental variables, deadwood characteristics, and tree species composition.

Diagonal panels show variable distributions; lower panels depict pairwise scatterplots, and upper panels display Pearson correlation coefficients with significance levels (p < 0.05, p < 0.01, p < 0.001). This visualization allows assessment of both linear associations and data distributions among key habitat features.


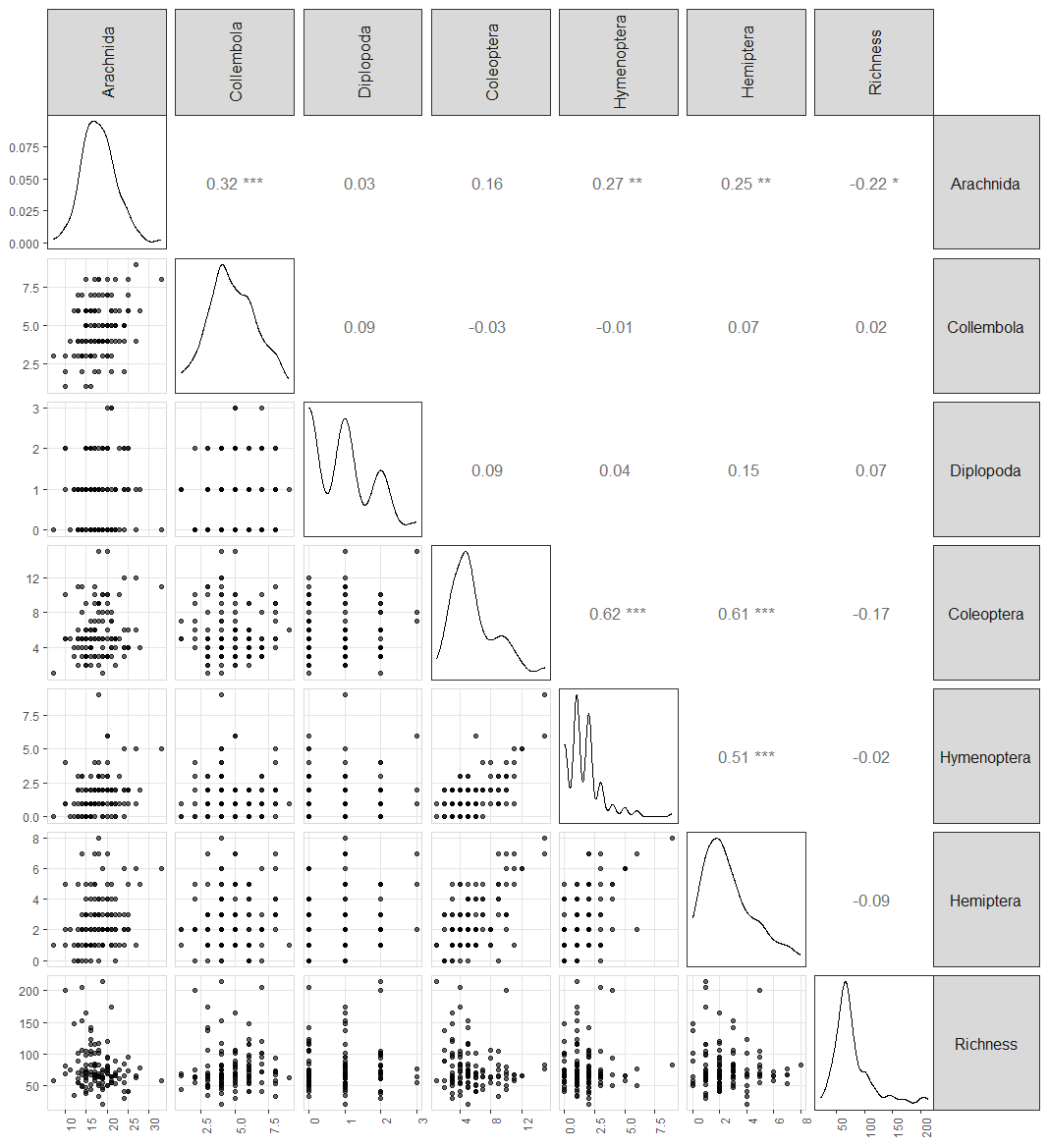


Figure S3. Scatterplot matrix showing pairwise relationships among arthropod taxonomic groups OTUs richness and overall species richness across all samples.

Diagonal panels display variable distributions; lower panels show scatterplots between groups, and upper panels indicate Pearson correlation coefficients with significance levels (p < 0.05, p < 0.01, p < 0.001).
