# Supplementary material for: Influence of Deadwood, Tree‐Related Microhabitats, and Forest Structural Features on Saproxylic Arthropod Diversity
Source: Ecol Evol. 2026 Apr 30;16(5):e73600. doi: 10.1002/ece3.73600 (PMC13129597; doi:10.1002/ece3.73600)
Supplement: Supplementary file 2 — Table S1: Frequency distribution of study plots across forest types and management categories. Forest types were classified based on the proportion of coniferous trees per plot (coniferous ≥ 80%, deciduous ≤ 20%, mixed 20%–80%). Management categories were derived from the Forest Management Intensity index (ForMI; low < 0.33, medium 0.33–0.66, high > 0.66). Table S2: Comprehensive overview of the identified tree‐related microhabitat (TreMs) associated arthropods at the taxonomic order, family, and species levels. [file ECE3-16-e73600-s001.docx]

**Supplements**

Table S1. Frequency distribution of study plots across forest types and management categories. Forest types were classified based on the proportion of coniferous trees per plot (coniferous ≥80%, deciduous ≤20%, mixed 20–80%). Management categories were derived from the Forest Management Intensity index (ForMI; low <0.33, medium 0.33–0.66, high >0.66).

| Forest type | Management category | Number of plots |
| --- | --- | --- |
| Coniferous | High | 52 |
| Coniferous | Medium | 6 |
| Deciduous | High | 3 |
| Deciduous | Low | 1 |
| Mixed | High | 43 |
| Mixed | Medium | 9 |
| Mixed | Low | 2 |

Table S2. Comprehensive overview of the identified tree-related microhabitat (TreMs) associated arthropods at the taxonomic order, family, and species levels.

| **Class** | **Order** | **Family** |
| --- | --- | --- |
| Arachnida | Mesostigmata | Ameroseiidae |
|  |  | Ascidae |
|  |  | BlattisoCiidae |
|  |  | Digamasellidae |
|  |  | Laelapidae |
|  |  | Melicharidae |
|  |  | Parasitidae |
|  |  | Phytoseiidae |
|  |  | Sejidae |
|  |  | Trachytidae |
|  |  | Zerconidae |
|  | Sarcoptiformes | Punctoribatidae |
|  |  | Scheloribatidae |
|  |  | Tegoribatidae |
| Chilopoda | Lithobiomorpha | Lithobiidae |
|  | Scolopendromorpha | Cryptopidae |
| Collembola | Entomobryomorpha | Entomobryidae |
|  |  | Isotomidae |
|  |  | Orchesellidae |
|  |  | Tomoceridae |
|  | Neelipleona | Neelidae |
|  | Poduromorpha | Hypogastruridae |
|  |  | Neanuridae |
|  | Symphypleona | Dicyrtomidae |
|  |  | Katiannidae |
|  |  | Sminthuridae |
| Diplopoda | Chordeumatida | Chordeumatidae |
|  | Glomerida | Glomeridae |
|  | Julida | Blaniulidae |
|  | Polydesmida | Polydesmidae |
| Insecta | Coleoptera | Cerambycidae |
|  |  | Ciidae |
|  |  | Cleridae |
|  |  | Dermestidae |
|  |  | Lucanidae |
|  |  | Melandryidae |
|  |  | Ptinidae |
|  |  | Staphylinidae |
|  | Diptera | Anisopodidae |
|  |  | Dolichopodidae |
|  |  | Muscidae |
|  |  | Syrphidae |
|  |  | Tachinidae |
|  |  | Tipulidae |
|  | Hymenoptera | Braconidae |
|  |  | Ichneumonidae |
|  |  | Sphecidae |
|  | Lepidoptera | Oecophoridae |
|  |  | Sesiidae |
|  |  | Tineidae |
|  | Raphidioptera | Inocelliidae |
|  |  | Raphidiidae |
|  | Thysanoptera | Phlaeothripidae |
|  |  | Thripidae |
